# On the Pathology and Treatment of Cholera

**Published:** 1853

**Authors:** 


### *On the Pathology and Treatment of Cholera.*

The author arranged his observations under the following heads, "Manner of Studying Cholera;" Literary History and Pathology;" "Treatment;" "General Conclusions." He observed, that the majority of authors who have treated cholera have drawn their descriptions too exclusively from the more appalling forms of the disease; and sufficient attention has not been paid to those cases which, from being mild, or in some way favorably circumstanced, terminate in recovery, with little or no medical interference. Much is to be learned from a study of simple cases which have not been disturbed by treatment. In many diseases, as pneumonia, scarlatina, &c., there are a number of cases which recover almost spontaneously, while others are saved by the skilful use of remedies; and, on the other hand, not a few are tremendously rapid in their fatal career; and we often meet with the mildest and the most terrible cases recurring simultaneously or consecutively in the same house. Dr. Cormack then insisted on the necessity, in the study of an epidemic, of observing the character of all the diseases within a given district; and referred to the great benefit which might arise from the formation of district societies of medical observation. The author then entered on the question of pathology of cholera. From the writings of Alibert, Comparetti, and other old authors, and from the observations of Dr. Charles Bell, Dr. James Bird, and himself, he was led to consider cholera to be essentially a pernicious intermittent fever. He did not claim originality for this view, but believed that periodicity has long been recognized as an essential element of cholera, and that its treatment by antiperiodic remedies is of equal antiquity. He summed up his observations on the pathology of cholera in the folllowing conclusions;—

'1. Cholera is a fever, intimately related to those fevers which depend on malaria.

2. The intermittent or remittent type can be generally recognized in the milder, and also not unfrequently (though less distinctly) in the severer cases.

3. The stage of collapse ought to be considered as an aggravated cold stage of the paroxysm of a pernicious fever, which may spontaneously terminate in death or reaction.

4. The least dense portion of the blood has an excessive tendency to exude through the capillaries of the stomach and bowels, and pass from the body by vomit and stool.

5. The inspissated residual blood being unable to pass through the small pulmonary vessels, causes congestion of the lungs; and as speedy consequences of this condition, paralysis of the right side of the heart from over-distention, asphyxia, and other subordinate derangements of the vital actions.

6. Death may take place from, *a.* Asphyxia. *b.* Necræmia, with loss of the least dense portion of the blood by stool and vomit. *c.* Necræmia, without such loss of the least dense portion of the blood as can be discovered during life—the exeudation remaining within the stomach and intestines. *d.* Toxæmia, from absence or deficiency of sanguineous depuration. *e.* Inflammation of lungs or other organs supervening in convalescence. *f.* Debility. *g.* Gastro-enteris. *h.* Two or more of the above causes combined.

7. The anatomical lesions found on dissection vary with the causes of, and circumstances attending death."

In the treatment of cholera, Dr. Cormack observed that it was of importance to bear in mind its relation to ague. Hence, in the first step, preparations of iron and quinine are of signal benefit. But we must, at the same time, if the digestive functions be at fault, employ a judicious alterative treatment; and if there should be a tendency to copious watery evacuations, we must restrain them by sulphuric acid, acetate of lead, creosote, nitrate of silver, or other hæmostatics. If the case has proceeded further, and it becomes necessary to treat the cramps and collapse, a solution of camphor in chloroform will be found most useful; and external warmth and stimulating embrocations are at the same time very beneficial.

Mr. Ross having made some observations,

Mr. Dendy eulogized the paper just read by Dr. Cormack, but differed in opinion with him respecting the identity of diarrhœa and cholera. He thought this a most fatal mistake, and that the diseases were entirely distinct. Cholera might come on without any diarrhœa being present, and diarrhœa might be cured and arrested without showing any sign of cholera. He thought he had got to the root of the disease when he considered it as a type of intermittent fever. He did not, however, agree with the recommendation that had been made of employing external heat in the cold stage; for he was sure that in the first epidemic, in 1832, he had seen people boiled and baked to death by such applications. He had tried the plan of external warmth in an infirmary with which he was then connected, but abandoned it from its disastrous results. He subsequently opened the windows of the ward, of course for the purpose of bringing on reaction, and sponged the body with cold water and vinegar, followed by frictions of salt-and-water, and then placed the patient in warm blankets. This plan had been most beneficial. Where no diarrhœa existed in cases of cholera, the whole intestinal tube after death was found full of serum, the flux having taken place into the internal cavities of the body.

Mr. Robinson, after speaking of the grave importance of the subject under inquiry, said that he regarded the disease as a poison in the system. In some cases, the poison might not be sufficient to kill, and the patients might recover without remedies being applied. But our inquiry, to be of service, should determine, if possible, what was the remedy which would be of most service in the greatest number of cases. It was true that under the most judicious treatment, many would die from the mere strength of the poison; but still, remedies might do good. What were those remedies? Now the dangers from cholera were two-fold: first, there was danger from collapse; and secondly, there was the danger from the after consequences, which were the result of toxæmia. He believed cholera might exist without any diarrhœa. He had the greatest faith in mercury, the action of which he thought was two-fold. First, it seemed to exert a direct action upon the poison itself; and secondly, it opened the gall ducts, and thus, by the free liberation of the bile, saved life. In all cases of recovery which he had seen, large quantities of vitiated bile passed from the intestines. He had seen in fatal cases the gall-bladder full of bile.

Dr. Crisp thought that the analogy drawn between ague and cholera by the author of the paper did not hold, for cholera neither resembled ague in its symptoms nor its topography. The symptoms were not of an intermittent character, and in many districts where ague prevailed not a single case of cholera had appeared. Moreover, cholera had often affected the inhabitants only of one side of a street or river, but no such case was observed respecting ague. Dr. Crisp differed from Dr. Cormack also in believing that Cholera had been described by the old writers. He believed it was a disease *sui generis*, and had never been seen before 1871, when the first case appeared at Jessore. Dr. Cormack spoke of the advantage of forming societies for the investigation of the disease, but Dr. Crisp thought that more good would be accomplished by carrying out the plan he had suggested in the paper alluded to—viz., "the appropriation of three wards in each of the large hospitals for the exclusive treatment of cholera, under the supervision of a government inspector." Three of the most approved modes of treatment might be tried, and in this way practical deductions come to. In estimating the value of medicines, it was most important to bear in mind the period of the epidemic when they were administered, the non-observance of this rule having led to many false inferences. Our treatment might be called empirical, but those who gave calomel had at least a motive for what they did; they believed that it served to stimulate the sluggish liver, and promote the secretion of bile. The advocates of sulphur supposed that this medicine destroyed or counteracted the effects of the poison that occasioned the disease; whilst those who recommended saline injections into the veins did so under the belief that it was necessary to restore the watery part of the blood. Dr. Crisp could see nothing empirical or unphilosophical in any of these modes of treatment. If no progress had been made in the treatment of cholera, much good had resulted from the investigation of some points connected with the disease, especially that of inspection. In 1831, the Board of Health, with the President of the College of Physicians at its head, recommended "that all articles of food should be placed in front of the house, and taken in when the person carrying them shall have retired;" "that the dead should be buried in detached ground, and troops and police placed around the infected districts," &c., &c. In the document, however, recently put forth to the public by the College of Physicians, it is stated, "that from the reports made to them the fear of infection may be practically disregarded." Another good had resulted from statistical investigations. In 1849, the authorities of Guy's Hospital, through fear of contagion, closed their gates against cholera patients, although hundreds were dying around; but now more cases of cholera had been admitted into this than into any other hospital. Dr. Crisp thought the statement respecting the comparative exemption of workers in metal from cholera was erroneous. In the statistics which he had formerly brought before the Society, out of 4258 males over twenty years of age dying in London during seventeen weeks in 1849, 102 were workers in iron, 25 in silver and gold, 30 in brass and copper, and 9 in tin; so that if the number of the various trades was considered, it would probably be found that the workers in metal enjoyed no peculiar exemption.

Dr. Webster quoted several authors to show that cholera was well known to the ancients, and mentioned more particularly the works of Dr. Morton, written in the last century, to show that cholera was not, as some supposed, a disease which had its origin in 1817. With respect to the objection raised by Dr. Crisp, that cholera and ague were not identical, on the ground that ague did not select one side of a river or one side of a street as cholera did, Dr. Webster remarked that in Rome it was not uncommon for the ague to be prevalent on one side of the Tiber, and also on one side of a street, whilst the other sides were free. With respect to treatment, he agreed with Dr. Cormack as to the efficacy of the exhibition of camphor and the application of external warmth. He also considered opium in amall doses, when joined with camphor, of great benefit. With respect to the contagious nature of cholera, his own opinion was that it was not contagious, and he instanced the facts that when the disease was most prevalent in Paris, no case occurred at Versailles, although there was constant railway communication between the places, whilst the disease spread in Sweden in spite of the strictest quarantine.

